# Estimating the Reproduction Number of Ebola Virus (EBOV) During the 2014 Outbreak in West Africa

**DOI:** 10.1371/currents.outbreaks.91afb5e0f279e7f29e7056095255b288

**Published:** 2014-09-02

**Authors:** Christian L. Althaus

**Affiliations:** Institute of Social and Preventive Medicine (ISPM), University of Bern, Bern, Switzerland

## Abstract

The 2014 Ebola virus (EBOV) outbreak in West Africa is the largest outbreak of the genus Ebolavirus to date. To better understand the spread of infection in the affected countries, it is crucial to know the number of secondary cases generated by an infected index case in the absence and presence of control measures, i.e., the basic and effective reproduction number. In this study, I describe the EBOV epidemic using an SEIR (susceptible-exposed-infectious-recovered) model and fit the model to the most recent reported data of infected cases and deaths in Guinea, Sierra Leone and Liberia. The maximum likelihood estimates of the basic reproduction number are 1.51 (95% confidence interval [CI]: 1.50-1.52) for Guinea, 2.53 (95% CI: 2.41-2.67) for Sierra Leone and 1.59 (95% CI: 1.57-1.60) for Liberia. The model indicates that in Guinea and Sierra Leone the effective reproduction number might have dropped to around unity by the end of May and July 2014, respectively. In Liberia, however, the model estimates no decline in the effective reproduction number by end-August 2014. This suggests that control efforts in Liberia need to be improved substantially in order to stop the current outbreak.

## Introduction

The 2014 Ebola virus (EBOV) outbreak in West Africa is the largest outbreak of the genus *Ebolavirus* to date. The outbreak began in Guinea in December 2013^1^ and later spread to Sierra Leone, Liberia and Nigeria. While public health interventions have been introduced in all affected countries, the numbers of infected cases and deaths from EBOV continue to increase and the effects of control measures remain to be determined. Real-time analysis of the numbers of infected cases and deaths due to EBOV could provide helpful information for public health policy.

Two key parameters describing the spread of an infection are the basic and the effective reproduction numbers, *R*_0_ and *R_e_*, which are defined as the number of secondary infections generated by an infected index case in the absence and presence of control interventions. If *R_e_* drops below unity, the epidemic eventually stops. Several studies have fitted mathematical models to data from previous outbreaks of the genus *Ebolavirus*.^2^^,^ 3 ^,^ 4 Previous estimates of *R*_0_ from two outbreaks in Congo (1995) and Uganda (2000) range from 1.3^2^ to 2.7.^4^ It will be important to know the reproduction numbers of the current EBOV outbreak and how it is affected by public health interventions. This will facilitate making projections of the epidemic during the next months and will allow comparisons of the effects of control measures in each country.

In this study, I describe the 2014 EBOV epidemic using an SEIR (susceptible-exposed-infectious-recovered) model. Fitting the model to the most recent data about reported cases and deaths in Guinea, Sierra Leone and Liberia provided estimates of the reproduction numbers of EBOV in absence and presence of control interventions.

## Methods

The transmission of EBOV follows SEIR (susceptible-exposed-infectious-recovered) dynamics and can be described by the following set of ordinary differential equations (ODEs):^2^

\begin{equation*}\frac{dS}{dt} = - \beta(t) S I/N,\\\%0A\frac{dE}{dt} = \beta(t) S I/N - \sigma E,\\\%0A\frac{dI}{dt} = \sigma E - \gamma I,\\\%0A\frac{dR}{dt} = (1-f)\gamma I.\end{equation*}

After transmission of the virus, susceptible individuals *S* enter the exposed class *E* before they become infectious individuals *I* that either recover and survive (*R*) or die. 1/*σ* and 1/*γ* are the average durations of incubation and infectiousness. The case fatality rate is given by *f*. The transmission rate in absence of control interventions is constant, i.e., *β*(*t*) = *β*. After control measures are introduced at time *τ* ≤ *t*, the transmission rate was assumed to decay exponentially at rate *k*:^3^

\begin{equation*}\beta(t) = \beta e^{-k(t-\tau)} ,\end{equation*}

i.e., the time until the transmission rate is at 50% of its initial level is *t*_1/2 _= ln(2)/*k*. Assuming the epidemic starts with a single infected case (*I*_0_ = 1 and *C*_0_ = 1), the cumulative number of infected cases *C* and deaths *D* are given by the solutions to *dC/dt* = *σE* and *dD/dt* = *fγI*, respectively. The ODEs were solved numerically in the R software environment for statistical computing^6^ using the function *ode* from the package *deSolve*.

Outbreak data for Guinea, Sierra Leone and Liberia (Table 1) were based on the cumulative numbers of reported total cases (confirmed, probable and suspected) and deaths from the World Health Organisation (WHO).^5^ The total population size *N = S + E + I + R* in each country was assumed to be 10^6^ individuals. Note that the exact population size does not need to be known to estimate the model parameters as long as the number of cases is small compared to the total population size.^2^ In particular, the basic reproduction number is simply given by *R*_0_ *= β*/*γ*. The effective reproduction number is given by** R_e_** = *β(t)S* **/(** **γN) ≈** *β(t)* */* *γ* as long as the number of cases remains much smaller than *S*.

Maximum likelihood estimates of the parameters were obtained by fitting the model to the data, assuming the cumulative numbers of cases and deaths are Poisson distributed. I used the optimization algorithm by Nelder & Mead that is implemented in the function *optim*. 95% confidence intervals (CI) were calculated from the likelihood profile.

**Table 1. Reported cumulative numbers of cases and deaths of EBOV during the 2014 outbreak.** Data were retrieved from the WHO website and based on the cumulative total numbers of clinical cases (confirmed, probable and suspected).^5^ The first confirmed cases in Sierra Leone were reported on 27 May 2014. In Liberia, the cases that were reported on 16 June 2014 were the first new cases since 6 April 2014.

Date	Guinea		Sierra Leone		Liberia	
	**Cases**	**Deaths**	**Cases**	**Deaths**	**Cases**	**Deaths**
22 Mar 2014	49	29				
24 Mar 2014	86	59				
25 Mar 2014	86	60				
26 Mar 2014	86	62				
27 Mar 2014	103	66				
28 Mar 2014	112	70				
31 Mar 2014	122	80				
01 Apr 2014	127	83				
04 Apr 2014	143	86				
07 Apr 2014	151	95				
09 Apr 2014	158	101				
11 Apr 2014	159	106				
14 Apr 2014	168	108				
16 Apr 2014	197	122				
17 Apr 2014	203	129				
20 Apr 2014	208	136				
23 Apr 2014	218	141				
01 May 2014	226	149				
03 May 2014	231	155				
05 May 2014	235	157				
07 May 2014	236	158				
10 May 2014	233	157				
12 May 2014	248	171				
18 May 2014	253	176				
23 May 2014	258	174				
27 May 2014	281	186	16	5		
28 May 2014	291	193				
29 May 2014			50	6		
01 Jun 2014	328	208	79	6		
03 Jun 2014	344	215				
05 Jun 2014	351	226	81	6		
06 Jun 2014			89	7		
15 Jun 2014	394	263	95	46		
16 Jun 2014	398	264			33	24
17 Jun 2014			97	49		
20 Jun 2014	390	270	158	34		
22 Jun 2014					51	34
30 Jun 2014	413	303	239	99	107	65
02 Jul 2014	412	305	252	101	115	75
06 Jul 2014	408	307	305	127	131	84
08 Jul 2014	409	309	337	142	142	88
12 Jul 2014	406	304	386	194	172	105
14 Jul 2014	411	310	397	197	174	106
17 Jul 2014	410	310	442	206	196	116
20 Jul 2014	415	314	454	219	224	127
23 Jul 2014	427	319	525	224	249	129
27 Jul 2014	460	339	533	233	329	156
30 Jul 2014	472	346	574	252	391	227
01 Aug 2014	485	358	646	273	468	255
04 Aug 2014	495	363	691	286	516	282
06 Aug 2014	495	367	717	298	554	294
09 Aug 2014	506	373	730	315	599	323
11 Aug 2014	510	377	783	334	670	355
13 Aug 2014	519	380	810	348	786	413
15 Aug 2014					834	466
16 Aug 2014	543	394	848	365		
18 Aug 2014	579	396	907	374	972	576
20 Aug 2014	607	406	910	392	1082	624

## Results

The limited number of data items and the fact that the EBOV outbreak is ongoing prevent the estimation of all model paramters. Hence, I made three simplifying assumptions. First, the average duration of the incubation and infectious period were fixed to previous estimates from an outbreak of the same EBOV subtype in Congo in 1995 (1/*σ* = 5.3 days and 1/*γ* = 5.61 days).^2^ Second, I assumed that control measures began after the appearance of the first infected case, i.e., *τ* = 0. Third, the date of appearance of the primary case of the outbreak in Guinea was set to 2 December 2013.^1^ The remaining parameters to be estimated are the transmission rate *β*, the case fatality rate *f*, the rate *k* at which control measures reduce the transmission rate and the time of appearance of the primary case that caused the subsequent outbreak *T* (for Sierra Leone and Liberia only).

The model fits the reported data of cases and deaths in Guinea, Sierra Leone and Liberia well (Fig. 1). The maximum likelihood estimates of the basic reproduction number, *R*_0_, are 1.51 (95% CI: 1.50-1.52) for Guinea, 2.53 (95% CI: 2.41-2.67) for Sierra Leone and 1.59 (95% CI: 1.57-1.60) for Liberia (Table 2). The case fatality rate *f* is estimated at 74% (95% CI: 72%-75%) for Guinea, 48% (95% CI: 47%-50%) for Sierra Leone and 71% (95% CI: 69%-74%) for Liberia. The estimates of the parameter *k*, which describes how the control measures reduce the transmission rate, vary between countries (Table 2). This results in a different decrease of the effective reproduction number, *R_e_*, after the outbreaks started in each country. While *R_e_* seems to have dropped to levels around unity in Guinea and Sierra Leone by end-August 2014, the model suggests that control interventions were not successful in reducing *R_e_* in Liberia (Fig. 2).

**Dynamics of 2014 EBOV outbreaks in Guinea, Sierra Leone and Liberia.**
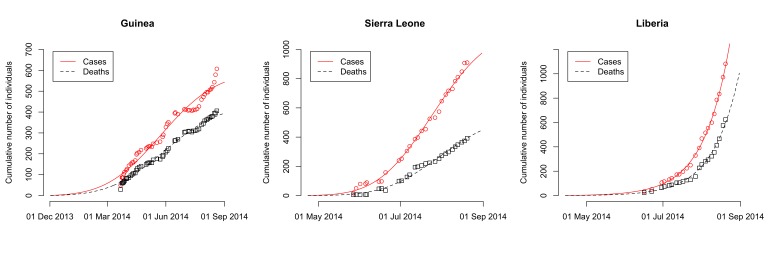
Data of the cumulative numbers of infected cases and deaths are shown as red circles and black squares, respectively. The lines represent the best-fit model to the data. Note that the scale of the axes differ between countries.

**Table 2. Parameter estimates for the 2014 EBOV outbreak.** The basic reproduction number is given by *R*_0_ = *β*/*γ* where 1/*γ* = 5.61 days is the infectious duration from the study by Chowell et al.^2^ The primary case is defined as the index patient that caused the subsequent outbreak. The date of appearance of the primary case in Guinea was set at 2 December 2013.^1^ 95% confidence intervals (CI) are shown in brackets. *A Likelihood ratio test showed that treating *k* as a free parameter does not improve the fit.

Parameter	Guinea	Sierra Leone	Liberia
Basic reproduction number, *R*_0_	1.51 (1.50-1.52)	2.53 (2.41-2.67)	1.59 (1.57-1.60)
Transmission rate, *β* (per day)	0.27 (0.27-0.27)	0.45 (0.43-0.48)	0.28 (0.28-0.29)
Case fatality rate, *f*	0.74 (0.72-0.75)	0.48 (0.47-0.50)	0.71 (0.69-0.74)
Rate at which control measures reduce transmission, *k* (per day)**	0.0023 (0.0023-0.0024)	0.0097 (0.0085-0.0110)	0*
Date of appearance of primary case, *T*	-	23 Apr 2014 (19-25 Apr 2014)	14 April 2014 (11-16 Apr 2014)

**Effective reproduction number of EBOV in Guinea, Sierra Leone and Liberia.**
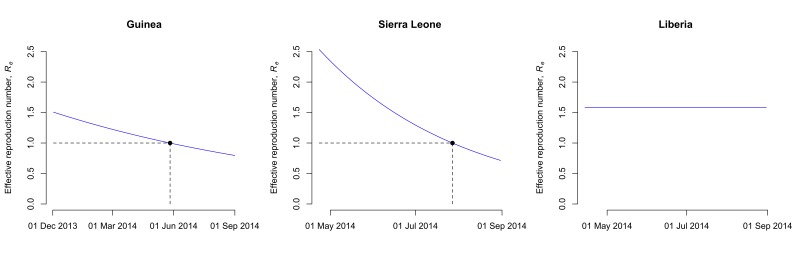
The model assumes that the transmission rate decays exponentially due to the introduction of control measures. In Guinea and Sierra Leone, the effective reproduction number has dropped to around unity by the end of May and July 2014, respectively (dashed lines). In Liberia, the effective reproduction number remains unchanged by end-August 2014. Note that the scale of the x-axis differs between countries.

## Discussion

This study uses mathematical modeling to estimate the basic and effective reproduction numbers of EBOV during the 2014 outbreak in West Africa. The maximum likelihood esitmates of *R*_0_ are 1.51 for Guinea, 2.53 for Sierra Leone and 1.59 for Liberia and lie within the same range as previous estimates for an EBOV outbreak in Congo (1995) and an outbreak of Sudan virus (SUDV) in Uganda (2000)**.** 2 ^,^ 3 ^,^ 4 The basic reproduction number in Sierra Leone seems to be significantly higher than in Guinea and Liberia. This could be a result of differences in the population structure or human mobility.

This study provides real-time estimates of EBOV transmission parameters during an ongoing outbreak. The mathematical model of transmission is informed by previous studies^2^^,^ 3 ^,^ 4and provides a good description of the current epidemic. Interestingly, the estimated date of appearance of the primary case that caused the subsequent outbreak in Sierra Leone is in agreement with the time of the most recent common ancestor of a genome analysis of the EBOV outbreak.^7^

The simplifying assumptions of the model mean that the results of this study need to be interpreted with caution. A major limitation of the model is that the transmission rate decays exponentially due to control measures after the appearance of the first infectious case. Thus, the model cannot account for fluctuations in the number of new cases as seen in Guinea. These fluctuations could be a result of varying effects of control interventions in different areas, suggesting that *R_e_* in some parts such as urban areas might well be above unity. As more data of the EBOV outbreak is becoming available, this modeling aspect might fit better to data from specific areas within a country. The effectiveness of current control measures on the transmission rate is unknown at present and the size and duration of the outbreak suggest that they need to improve. As more data accumulate over the next weeks and months a more thorough analysis will allow more accurate estimates of *R*_0_ and *R*_e_ together with epidemic projections and the uncertainty around them.

Real-time estimates of epidemic parameters and model projections are important for predicting the evolution of this EBOV epidemic and investigating the effects of ongoing and new interventions. Hygiene measures and social distancing intervention are already being implemented and WHO has given permission for the use and evaluation of currently unregistered new drugs and vaccines. The results of this study suggest that control measures might have been sufficient to decrease the effective reproduction number to around unity in Guinea and Sierra Leone by the end of May and July 2014, respectively. In Liberia, however, control efforts still need to be improved substantially in order to stop the current outbreak.

## Competing Interests

The author has declared that no competing interests exist.

## References

[1] Baize S, Pannetier D, Oestereich L, Rieger T, Koivogui L, et al. (2014). Emergence of Zaire Ebola Virus Disease in Guinea - Preliminary Report. N Engl J Med. 10.1056/NEJMoa1404505 24738640

[2] Chowell G, Hengartner NW, Castillo-Chavez C, Fenimore PW, Hyman JM. (2004)The basic reproductive number of Ebola and the effects of public health measures: the cases of Congo and Uganda. J Theor Biol, 229(1):119–26. 10.1016/j.jtbi.2004.03.006 15178190

[3] Lekone PE, Finkenstädt BF. (2006). Statistical inference in a stochastic epidemic SEIR model with control intervention: Ebola as a case study. Biometrics, 62(4):1170– 7. 10.1111/j.1541-0420.2006.00609.x 17156292

[4] Legrand J, Grais RF, Boelle PY, Valleron AJ, Flahault A. (2007). Understanding the dynamics of Ebola epidemics. Epidemiol Infect, 135(4):610–21. 10.1017/S0950268806007217 PMC2870608 16999875

[5] Disease Outbreak News - WHO | Regional Office for Africa

[6] R Development Core Team (2014). R: A Language and Environment for Statistical Computing. R Foundation for Statistical Computing, Vienna, Austria.

[7] Gire SK, Goba A, Andersen KG, Sealfon RS, Park DJ, et al. (2014). Genomic surveillance elucidates Ebola virus origin and transmission during the 2014 outbreak. Science, published online 28 August 2014. 10.1126/science.1259657 PMC4431643 25214632

